# Psychometric Properties of Three Simplified Chinese Online-Related Addictive Behavior Instruments Among Mainland Chinese Primary School Students

**DOI:** 10.3389/fpsyt.2020.00875

**Published:** 2020-09-03

**Authors:** I-Hua Chen, Daniel Kwasi Ahorsu, Amir H. Pakpour, Mark D. Griffiths, Chung-Ying Lin, Chao-Ying Chen

**Affiliations:** ^1^ Chinese Academy of Education Big Data, Qufu Normal University, Shandong, China; ^2^ School of Education Science, Minnan Normal University, Zhangzhou, China; ^3^ Department of Rehabilitation Sciences, Faculty of Health and Social Sciences, The Hong Kong Polytechnic University, Hong Kong, Hong Kong; ^4^ Social Determinants of Health Research Center, Research Institute for Prevention of Non-Communicable Diseases, Qazvin University of Medical Sciences, Qazvin, Iran; ^5^ Department of Nursing, School of Health and Welfare, Jönköping University, Jönköping, Sweden; ^6^ International Gaming Research Unit, Psychology Department, Nottingham Trent University, Nottingham, United Kingdom; ^7^ Institute of Allied Health Sciences, College of Medicine, National Cheng Kung University, Tainan, Taiwan

**Keywords:** child technology use, gaming addiction, psychometrics, social media addiction, smartphone addiction

## Abstract

**Background/Objective:**

There are inadequate screening instruments for assessing specific internet-related addictions among mainland Chinese primary school students. Therefore, the present study validated the psychometric properties of three simplified Chinese online-related addictive behavior instruments among mainland Chinese primary school students.

**Method:**

Fourth to sixth graders (n = 1108; 48.3% males; mean [SD] age = 10.37 years [0.95]) completed the nine-item Internet Gaming Disorder Scales-Short Form (IGDS-SF9), Bergen Social Media Addiction Scale (BSMAS), and Smartphone Application-Based Addiction Scale (SABAS) in a classroom. The factorial structures and the unidimensionality of the three scales were examined using confirmatory factor analyses (CFAs). Measurement invariance of the three scales was examined using multigroup confirmatory factor analyses (MGCFAs) across gender.

**Results:**

The findings demonstrated that the three scales (Cronbach’s *α* = 0.73 to 0.84) had unidimensional structure as supported by satisfactory fit indices (comparative fit index = 0.98 to 1.00). The MGCFA findings indicated that the unidimensional structures of the three scales were invariant across gender.

**Conclusions:**

The findings indicate that the three simplified Chinese scales (IGDS-SF9, BSMAS, and SABAS) are valid instruments for assessing online-related addictive behaviors among mainland Chinese primary school students irrespective of their gender.

## Highlights

The present study tested the appropriateness of three online-related addictive behavior instruments (*i.e.*, Internet Gaming Disorder Scales-Short Form [IGDS-SF9], Bergen Social Media Addiction Scale [BSMAS], and Smartphone Application-Based Addiction Scale [SABAS]), and they were found to be suitable for use among Chinese children. These instruments will be of importance to researchers and clinicians as they may help ascertain the risk of online-related addictions among these children.

## Introduction

Internet use has become an important part in individuals’ daily lives, especially during the past decade given the rapid growth of modern technology. With the emergence of new technologies, internet accessibility and the availability *via* Wi-Fi enabled devices such as smartphones, tablets, laptops, and desk computers have become widespread. This technological advancement has eliminated geographical barriers between people by facilitating communication, business transactions, online games, online shopping, and other forms of entertainment (1). While there are many benefits associated with internet use (*e.g.*, communication, information searching, and entertainment), use of such technology may lead to mental health challenges such as addiction, alexithymia, and suicide risk (2, 3) for a small minority of individuals if not properly managed.

Internet-related addiction has been a notable problem among young people and increasingly among children given that children are now being given access to tablets and smartphones (4). Internet-related addiction basically involves problematic overuse of the internet for various activities such as online gaming, chatting (*e.g.*, *Facebook, Twitter, QQ*, and *Wechat*), and gambling purposes (5). For some individuals, such internet-related activities may seem entertaining and benign at first. However, with poor self-control, it may become an addictive behavior and may subsequently lead to serious and negative psychosocial effects (*e.g.*, preoccupation and cognitive distortions, worsening social behaviors, and unpleasant feelings or physical discomfort) (6). In addition, there are significant associations between internet-related addiction and poor mental health (*e.g.*, depression, anxiety, and stress) (7–9). It has further been reported that in China (where the present study was carried out) there is a pooled internet addiction prevalence rate of 7.5% among Chinese adolescents (10) and approximately 11% among college students (11). These rates were higher in males than in females (11, 12). The relatively high prevalence rate in China demonstrates a worrisome situation. Therefore, there is a need for effective measures to prevent further internet addiction.

Notwithstanding these negative effects, apart from Internet Gaming Disorder (IGD) being in the appendix of the *Diagnostic and Statistical Manual of Mental Disorders*, fifth edition (DSM-5) (13), internet-related disorders are not officially recognized. Consequently, more studies are needed to establish the diagnostic criteria and course descriptions required to identify problematic internet-related behaviors as mental disorders (13). Since 2013, increasingly more studies have been conducted to identify these behaviors and to establish how to accurately assess and diagnose the subcategories of internet-related addictive disorders.

The broad content area of online-related addictive behaviors (*e.g.*, gaming, social media) has led several studies to focus on developing and/or standardizing scales that can reliably and validly assess an aspect of internet-related behavior (or disorder) to help establish diagnostic criteria [*e.g.*, Leung et al. (14); Yam et al. (15)]. Also, knowing that specific internet-related addictions such as smartphone addiction, social media addiction, and internet gaming disorder do not share similar features with generalized internet addiction (5, 16, 17), helps researchers and practitioners to better understand general and specific internet-related addictive behaviors so as to better diagnose and offer effective treatment strategies. Additionally, there is very limited research on understanding of addictive behaviors related to internet and smartphone use among mainland Chinese children (18–20), taking into consideration that China has one of the highest prevalence rates for internet-related addictions such as internet gaming (13). Therefore, there is a need to validate culturally-specific instruments that are brief enough to assess some specific internet-related addictions among mainland Chinese children. More specifically, the simplified Chinese versions of the nine-item Internet Gaming Disorder Scale (IGDS-SF9), the Bergen Social Media Addiction Scale (BSMAS), and the Smartphone Application-Based Addiction Scale (SABAS) were the main instruments of validation in this study. Consequently, the major aims of the present study were to translate these three internet-related instruments into simplified Chinese versions and to validate these translated versions among children and to examine its relationships with different types of psychological distress (*i.e.*, depression, anxiety, and stress).

## Methods

### Translation Procedure

Because the three instruments have already been translated into traditional Chinese character versions (14, 15, 21), these versions were used to create simplified Chinese character versions. More specifically, the first step was to convert every traditional Chinese character into its corresponding simplified Chinese character. The second step was to modify some terms to make the simplified Chinese character versions be better understood by mainland Chinese individuals. For example, mainland Chinese rarely use *Facebook* or *Twitter* (in fact, using *Facebook* or *Twitter* is not officially allowed in mainland China). Therefore, commonly used social media like *QQ* and *Wechat* were used instead in the simplified Chinese character versions. The final step was to ask five primary school teachers to evaluate the simplified Chinese character versions and to ensure their readability.

### Participants and Procedure

The study was approved by the ethics committee of the Hong Kong Polytechnic University (IRB ref: HSEARS20190718001) before the targeted participants (*i.e.*, primary school children in the fourth to sixth grades) were approached. Three primary schools in Sichuan were contacted and agreed to assist in this study. Teachers in the three schools further helped in distributing the study information to children in the fourth to sixth grades. After ensuring the willingness to participate from both the primary school students and their parents, several scales together with a background information sheet were given to the students to complete. Also, written informed consent was obtained for every participant (and one of their parents) who agreed to participate. The survey was conducted in the classroom under the supervision of the school teachers in the precise order outlined in the ‘*Measures*’ section. The inclusion criteria for participants were that they had to (i) understand written Chinese in simplified characters; (ii) understand spoken Mandarin; (iii) have had their smartphone with internet access over three months; and (iv) have the ability to complete the survey without difficulties. Out of 1,150 participants, 1,108 (96.35% response rate) participated in the present study. The participants did not receive any incentive; however, the present authors expressed appreciation to the participants.

### Measures

#### Internet Gaming Disorder Scale-Short Form (IGDS-SF9)

The nine-item IGDS-SF9 developed by Pontes and Griffiths (22) is a short self-report scale that is used to assess Internet Gaming Disorder based on DSM-5 criteria (13). It uses a five-point Likert-scale response format that ranges from 1 (*Never*) to 5 (*Very often*) with a higher sum of item scores indicating a greater degree of internet gaming disorder. An example item is *“Do you systematically fail when trying to control or cease your gaming activity?”*. The confirmatory factor analysis (CFA) of the IGDS-SF9 demonstrated a unidimensional structure with satisfactory psychometric properties which include internal consistency (Cronbach’s *α* of 0.9), criterion and construct validity especially among Hong Kong university students (15). It also has acceptable psychometric properties in different languages including English (22, 23), Persian (24), Portuguese (25), Spanish (26), Slovenian (27), Italian (28), Malaysian (29), and Turkish (30, 31), making it one of the best scales for assessing IGD.

#### Bergen Social Media Addiction Scale (BSMAS)

The BSMAS, developed by Andreassen et al. (32), is a six-item scale used to assess the risk of social media addiction based on the addiction component model (*i.e.*, salience, mood modification, tolerance, withdrawal conflict and relapse) (6, 33). It uses a five-point Likert-scale response format that ranges from 1 (*Very rarely*) to 5 (*Very often*) with a higher sum of item scores indicating a greater degree of social media addiction. An example item is *“How often during the last year have you felt an urge to use social media more and more?”*. A CFA of the BSMAS demonstrated a unidimensional structure with satisfactory psychometric properties which include internal consistency (Cronbach’s *α* of 0.82), criterion and construct validity among Hong Kong university students (15). A national representative study among 5,961 Hungarian adolescents proposed a cutoff score of 19 out of 30 to indicate problematic use of social media (34). It has acceptable psychometric properties in different languages including English (32), Persian (35), and Italian (36).

#### Smartphone Application-Based Addiction Scale (SABAS)

The SABAS, developed by Csibi, Demetrovics, and Szabo (35), is a six-item scale that is used to assess the risk of smartphone addiction and is also based on addiction components model (*i.e.*, salience, mood modification, tolerance, withdrawal conflict and relapse) (6, 33). Its items (*e.g.*, *“My smartphone is the most important thing in my life.”)* are on a six-point Likert-scale response format that ranges from 1 (*Strongly disagree*) to 5 (*Strongly agree*) with a higher sum of item scores indicating a greater degree of smartphone addiction. A CFA of SABAS demonstrated a unidimensional structure with satisfactory psychometric properties which include internal consistency (Cronbach’s *α* of 0.75), criterion and construct validity among Hong Kong university students (15). Furthermore, other studies reported acceptable psychometric properties in different languages which includes Persian (37), English (38), Turkish (39, 40), Italian (41), Serbian (42), and Hungarian (35).

#### Depression, Anxiety, Stress Scale-21 (DASS-21)

The DASS-21, developed by Lovibond and Lovibond (43), is a 21-item scale that is used to assess three types of psychological distress (*i.e.*, depression, anxiety, and stress). Each type of distress (depression, anxiety, and stress) is assessed by seven items rated on a four-point Likert scale response format ranging from 0 (*Did not apply to me at all*) to 3 (*Applied to me very much, or most of the time*). A higher sum of item score for each type of distress indicates that an individual suffers more from that specific distress. It has been reported to have high internal consistency indices among adolescents (44, 45).

### Data Analysis

The participants’ characteristics were analyzed using descriptive statistics, including means (and SDs) for continuous variables, and frequency (%) for categorical variables. The associations between the studied variables (including IGDS-SF9, BSMAS, SABAS, DASS-21, and time spent on gaming, social media, and smartphone) were analyzed using Pearson’s correlation. Psychometric properties of the three instruments in scale level were analyzed using floor and ceiling effects (acceptable effects were <20.0%) (46), internal consistency by Cronbach’s *α* (acceptable value was >.7) (47), and CFA. Psychometric properties of the three instruments in item-level were analyzed using corrected item-total correlation (acceptable correlation was >.4) (48) and factor loadings derived from CFA.

CFA with diagonally weighted least squares (DWLS) estimator was applied to test whether the three psychometric instruments were unidimensional, according to the literature (27, 36, 38). Several fit indices were accordingly used to evaluate the unidimensionality of the three instruments: a nonsignificant χ^2^, the comparative fit index (CFI) > 0.9, the root mean square error of approximation (RMSEA) < 0.08, and the standardized root mean square residual (SRMR) <0.08 (49, 50). After ensuring the unidimensionality of the three instruments, measurement invariance across gender was carried out to examine whether male and female primary school students interpreted the three instruments similarly. Multigroup CFA (MGCFA) was used by three nested models to determine whether measurement invariance was supported. The nested models were a configural model, a model with factor loadings constrained equal across gender, and a model with factor loadings and item thresholds constrained equal across gender (51, 52). Two comparisons were conducted in the nested models (*i.e.*, configural model *vs.* model with factor loadings constrained equal, and model with factor loadings constrained equal *vs.* model with factor loadings and item thresholds constrained equal) to examine whether measurement invariance was supported. The following indices were used for the evaluation: ΔCFI > −0.01 (53), ΔRMSEA < 0.02 (54), and ΔSRMR < 0.03 (for invariant loadings) or 0.01 (for invariant thresholds) (55). The analyses were performed using LISREL 8.8 (Scientific Software International, Lincolnwood, IL, USA) for CFA and MGCFA; IBM SPSS Statistic version 24.0 (IBM Corp., Armonk, NY) for the rest of the analyses.

## Results

**Table 1** shows the participant characteristics (N = 1,108) whose mean age was 10.37 years (SD = 0.95). Nearly half of the participants were males (48.3%), and the participants were roughly equally distributed in three grades (fourth graders = 30.9%; fifth graders = 35.5%; sixth graders = 32.3%). On average, the participants spent 1.11 h/day (SD = 1.64) on their smartphone, 0.40 h/day (SD = 0.89) on social media, and 0.76 h/day (SD = 1.52) on gaming. Additionally, their psychological distress scores were 2.51 (SD = 3.61) for depression; 3.92 (SD = 3.86) for anxiety; and 4.31 (SD = 4.10) for stress.

**Table 1:** Participant characteristics (n = 1,108).

	Mean (SD) or n (%)	Missing n
Age (Year)	10.37 (0.95)	46
Gender (male)	513 (48.3%)	9
Grade		15
Grade 4	328 (30.9%)	
Grade 5	377 (35.5%)	
Grade 6	343 (32.3%)	
Time on smartphone (hours per day)	1.11 (1.64)	27
Time on using social media (hours per day)	0.40 (0.89)	43
Time on gaming (hours per day)	0.76 (1.52)	38
Depression score^a^	2.51 (3.61)	94
Anxiety score^a^	3.92 (3.86)	82
Stress score^a^	4.31 (4.10)	94

a Assessed using the Depression, Anxiety, Stress Scale (DASS-21).

**Table 2** shows the satisfactory properties of the IGDS-SF9, BSMAS, and SABAS at the scale level among the primary school students, except for the slightly high proportion in floor effects (IGDS-SF9 = 24.6%; BSMAS = 28.2%; SABAS = 25.7%). More specifically, all the fit indices used in the CFA supported the unidimensional structure for IGDS-SF9, BSMAS, and SABAS. Additionally, Cronbach’s *α* was satisfactory in all three scales: *α* = 0.84 for IGDS-SF9; 0.73 for BSMAS; and 0.81 for SABAS. Moreover, the IGDS-SF9, BSMAS, and SABAS were mutually significantly correlated (r = 0.55–0.69; all *p*-values < 0.01). Scores on the subscales for depression (r = 0.34–0.55; *p*-values < 0.01), anxiety (r = 0.35–0.47; *p*-values < 0.01), and stress (r = 0.37–0.52; *p*-values < 0.01) were all significantly correlated to scores on the IGDS-SF9, BSMAS, and SABAS as well as the daily time spent on smartphone, social media and gaming (**Table 3**).

**Table 2:** Psychometric properties of the three scales in scale level (n = 1,108).

Psychometric testing	IGDS-SF9	BSMAS	SABAS	Suggested cutoff
Ceiling effects (%)	0	0	0	<20
Floor effects (%)	24.6	28.2	25.7	<20
Internal consistency (Cronbach’s *α*)	0.84	0.73	0.81	>0.7
Confirmatory factor analysis (CFA)				
χ^2^ (*df*)	200.71(27)	25.93 (9)	19.51 (9)	Nonsignificant
Comparative fit index	0.98	0.99	1.00	>0.9
Tucker-Lewis index	0.98	0.99	1.00	>0.9
RMSEA	0.076	0.041	0.032	<0.08
SRMR	0.067	0.033	0.023	<0.08

IGDS-SF9, Internet Gaming Disorder Scale–Short-Form; no modification indices were done in the CFA; BSMAS, Bergen Social Media Addiction Scale; no modification indices were done in the CFA; SABAS, Smartphone Application-Based Addiction Scale; no modification indices were done in the CFA; RMSEA, Root-mean square error of approximation; SRMR, Standardized root mean square residual.

**Table 3:** Correlation matrix among the studied factors (n = 1,065–1,108).

*r*									
	1.IGDS-SF9	2.BSMAS	3.SABAS	4.Depression	5.Anxiety	6.Stress	7.Time on smartphone	8.Time on social media	9.Time on gaming
1.	–								
2.	0.55	–							
3.	0.69	0.55	–						
4.	0.55	0.34	0.44	–					
5.	0.47	0.35	0.39	0.69	–				
6.	0.52	0.37	0.42	0.69	0.75	–			
7.	0.29	0.20	0.30	0.19	0.17	0.21	–		
8.	0.16	0.16	0.15	0.13	0.17	0.17	0.60	–	
9.	0.20	0.16	0.23	0.17	0.13	0.19	0.70	0.59	–

All p-values < 0.01.

Depression, Anxiety, and Stress were assessed using Depression, Anxiety, Stress Scale (DASS-21); IGDS-SF9, Internet Gaming Disorder Scale–Short-Form; BSMAS, Bergen Social Media Addiction Scale; SABAS, Smartphone Application-Based Addiction Scale.

The item properties were all satisfactory in the IGDS-SF9, BSMAS, and SABAS. In the IGDS-SF9, all factor loadings derived from the CFA were between 0.60 and 0.80; corrected item-total correlations between 0.55 and 0.76. In the BSMAS, all factor loadings derived from the CFA were between 0.59 and 0.73; corrected item-total correlations between 0.61 and 0.68. In the SABAS, all factor loadings derived from the CFA were between 0.63 and 0.80; corrected item-total correlations between 0.66 and 0.76 (**Table 4**).

**Table 4:** Item properties and internal consistency (n = 1,108).

Scale or Item #	Item description	Mean (SD)	Factor loading^a^	Item-total correlation
**IGD9-SF**				
I1	Preoccupied with gaming behavior	1.63 (0.88)	0.60	0.63
I2	Feel more irritability, anxiety when reduce	1.46 (0.86)	0.78	0.71
I3	Spend more time to achieve pleasure	1.61 (0.91)	0.80	0.76
I4	Systematically fail when trying to control gaming activity	1.61 (0.95)	0.64	0.63
I5	Lost interests in previous hobbies	1.39 (0.85)	0.75	0.69
I6	Continued your gaming activity despite knowing it was causing problems	1.35 (0.73)	0.77	0.68
I7	Deceived about the amount of gaming activity	1.22 (0.66)	0.74	0.60
I8	Temporarily escape or relieve a negative mood	1.79 (1.12)	0.68	0.69
I9	Jeopardized or lost an important relationship	1.27 (0.74)	0.70	0.55
**BSMAS**				
B1	Salience	1.62 (0.88)	0.73	0.68
B2	Craving/tolerance	1.67 (0.91)	0.70	0.67
B3	Mood modification	1.36 (0.75)	0.67	0.61
B4	Relapse/loss of control	1.74 (1.06)	0.65	0.68
B5	Withdrawal	1.65 (1.02)	0.68	0.68
B6	Conflict/functional impairment	1.56 (1.01)	0.59	0.61
**SABAS**				
S1	Most important thing	2.00 (1.32)	0.63	0.66
S2	Conflicts have arisen	1.63 (1.21)	0.77	0.70
S3	Preoccupying myself	2.15 (1.42)	0.69	0.71
S4	Fiddle around more	1.82 (1.24)	0.79	0.76
S5	Irritable	1.78 (1.30)	0.80	0.76
S6	Fail to use less	1.90 (1.41)	0.75	0.73

IGDS-SF9, Internet Gaming Disorder Scale–Short-Form; BSMAS, Bergen Social Media Addiction Scale; SABAS, Smartphone Application-Based Addiction Scale.

^a^Factor loadings were derived from confirmatory factor analysis.

After ensuring that both scale-level and item-level of the IGDS-SF9, BSMAS, and SABAS were satisfactory and all had a unidimensional structure, the MGCFAs were applied to evaluate whether male and female primary school students interpreted the scales differently. All three instruments had their measurement invariance supported at factor loadings and item thresholds without relaxing any parameters (**Table 5**).

**Table 5:** Measurement invariance of the IGDS-SF9, BSMAS, and SABAS across gender (n = 1,099).

	Configural model^a^	Loadings constrained equal^a^	Loadings and thresholds constrained equal^a^
**BSMAS**			
χ^2^ (*df*) or Δχ^2^ (Δ*df*)	33.16 (18)	8.30 (5)	5.16 (5)
*p*-value	0.016	0.011	0.015
CFI or ΔCFI	0.99	0.000	0.000
RMSEA or ΔRMSEA	0.039	−0.001	−0.003
SRMR or ΔSRMR	0.040	0.010	0.000
**SABAS**			
χ^2^ (*df*) or Δχ^2^ (Δ*df*)	32.43 (18)	5.80 (5)	4.37 (5)
*p*-value	0.019	0.024	0.038
CFI or ΔCFI	1.00	0.000	0.000
RMSEA or ΔRMSEA	0.038	−0.003	−0.004
SRMR or ΔSRMR	0.030	0.003	0.000
**IGDS-SF9**			
χ^2^ (*df*) or Δχ^2^ (Δ*df*)	199.61 (54)	21.15 (8)	19.24 (8)
*p*-value	<0.001	<0.001	<0.001
CFI or ΔCFI	0.98	0.000	0.000
RMSEA or ΔRMSEA	0.070	−0.002	−0.001
SRMR or ΔSRMR	0.053	0.003	0.000

BSMAS, Bergen Social Media Addiction Scale; SABAS, Smartphone Application-Based Addiction Scale; IGDS-SF9, Internet Gaming Disorder Scale-Short Form; CFI, comparative fit index; RMSEA, root mean square error of approximation; SRMR, standardized root mean square residual; ^a^Configural models are reported using χ^2^ (df), CFI, RMSEA, and SRMR; other models are reported using Δχ^2^ (Δdf), ΔCFI, ΔRMSEA, and ΔSRMR.

## Discussion

The present study validated three instruments that are brief enough to assess some specific (*i.e.*, IGDS-SD9 and BSMAS) or general (*i.e.*, SABAS) internet-related addictions among mainland Chinese children. The results demonstrated that the IGDS-SF9, BSMAS, and SABAS all had robust psychometric properties among mainland Chinese primary school students. Furthermore, all three scales demonstrated a unidimensional structure which is consistent with previous studies (14, 15). Moreover, there were significant intercorrelations between all the variables including IGDS-SF9, BSMAS, SABAS, DASS-21 subscales, and the time spent on gaming, social media, and smartphones.

The results for IGDS-SF9 specifically showed that the scale had a unidimensional structure with acceptable internal consistency (*α* = 0.84). This indicates that IGDS-SF9 is reliable in assessing the risk of IGD among primary school students. These findings (unidimensional structure and reliability) are similar to previous studies [*e.g.*, Arıcak et al. (30); Beranuy et al. (26); Monacis et al. (28); Pontes & Griffiths (22); Pontes et al. (25, 27); T’ng & Pau (29); Wu et al. (24); Yam et al. (15)]. Also, the floor effect (24.6%) was higher than the suggested cut-off although similar to a study by Yam et al. (15). This is possible because the students spent little time on internet gaming. Indeed, the present study’s sample spent approximately 0.76 hours per day playing games, a shorter duration as compared with other studies (mean hours per day = 1.09 to 10.99) (15, 24, 56). The reasons for so little time spent on internet gaming may include parental restrictions on the use of gaming devices (and the like) as children. However, this floor effect finding is contrary to a study which found low floor and ceiling effects for quality of life instruments among children (57). Therefore, a possible reason for the higher floor effect in the present study as compared with the quality of life instruments (57) may be due to the difficulty accessing technology among children. Additionally, measurement invariance across gender was supported for IGDS-SF9, indicating that both genders had similar interpretations on the item content in the IGDS-SF9.

The findings for BSMAS also indicated that the scale had a unidimensional structure with acceptable internal consistency (*α* = 0.73). This indicates that the BSMAS is reliable in assessing the students’ challenges with social media use. These findings (unidimensional structure and reliability) are similar to previous studies (15, 36, 58). Results also showed the floor effect (28.2%) was higher than the suggested cut-off and that in previous studies (15, 35). This may be because the students spent little time on social media. Indeed, the present study’s sample spent about 0.40 h per day on social media, a shorter duration as compared with past studies (mean hours per day = 3.11 to 3.75) (15, 58). This finding is contrary to a study by Lin et al. (57) which reported low floor and ceiling effects for quality of life instruments among children. Similar to the explanation for the IGDS-SF9, the higher floor effect in the present study as compared with the quality of life instruments (57) may be due to the difficulty accessing technology among children. In addition, measurement invariance across genders was supported for the BSMAS, indicating that both genders had similar interpretations on the item content in the BSMAS (58).

The SABAS had similar psychometric properties like IGDS-SF9 and BSMAS. The scale had a unidimensional structure with acceptable internal consistency (*α* = 0.81). This indicates that the SABAS is reliable in assessing the students’ challenges with smartphone use. These findings (unidimensional structure and reliability) are similar to previous studies (15, 35, 37–39, 41). Also, the floor effect (25.7%) was higher than the suggested cut-off and that in previous studies (15, 37, 57). This may be possible because the students had low levels of smartphone use. The present study’s sample spent about 1.11 h per day on smartphones, a shorter duration as compared with previous research (mean hours per day = 5.29) (15). This may have accounted for the increased floor effect because they did not use smartphones excessively. Nonetheless, the ceiling effects were as anticipated. In addition, measurement invariance across genders was supported for the SABAS, indicating that both genders had similar interpretations on the item content in the SABAS.

In addition, there were significant positive relationships between the three scales (IGDS-SF9, BSMAS, and SABAS) and psychological distress (depression, anxiety, stress; r = 0.34–0.55), as well as interrelationships between IGDS-SF9, BSMAS, and SABAS (r = 0.55–0.69). These results confirm the criterion validity of IGDS-SF9, BSMAS, and SABAS. Also, the results re-affirm the association between individuals who are addicted to online-related activities and possibilities of them also having psychological distress (*i.e.*, depression, anxiety, and stress). Although the present study was unable to establish cause-and-effect relationship, possible reasons for these associations among heavy-using children may be that they are using online-related activities as a coping mechanism or they encounter challenges with the use of digital devices. This is similar to other studies (9, 15) except that the students used in this present study were children.

This study has practical implications for researchers and clinicians. The results indicate that future researchers in China may use these three scales for further studies of addiction risk to internet-related activities among Chinese children. Clinicians may also use it to assess the risk of online-related addictive behaviors among children. Although these scales are acceptable for assessing the risk of online-related addictive behaviors, they cannot be used as diagnostic tools. Consequently, further studies may be needed to help develop diagnostic criteria (or clinical cut-off scores) for the online-related behaviors examined in the present study.

The present study also has some limitations that should be considered when interpreting the findings. First, the study used mainland Chinese children. Therefore, the results cannot be generalized to other age groups. Moreover, most of the participants in the present study were Han Chinese. Therefore, the psychometric findings in the present study may not be able to generalize to other ethnic groups in mainland China. It is therefore recommended that this study be replicated in other ethnicities and age groups in China. Second, the study utilized self-reported instruments, and there is the chance that the findings may have been affected by social desirability biases or poor memory recall, especially given that children’s cognition is still in development. Third, the study did not collect data on participants’ psychiatric history which has the potential of confounding study findings. Fourth, the study did not examine the effect that the length of time that the student had owned a smartphone might have had on the results. This may have contributed to the slightly high floor effect found in the study. Future studies are thus needed to examine whether the length of time a student has owned a smartphone contributes to the high floor effect for the instruments. Future studies should take the recommendations into consideration when replicating this study. More specifically, future studies may consider using diagnostic measures (*e.g.*, diagnosis using DSM-5 or Structured Clinical Interview for DSM-*5* [SCID-5]) to examine the psychometric properties of the three online-related addiction behavior instruments. Also, respondents’ psychiatric history should be collected in the future study to avoid the influence of inherent confounding psychiatric variables.

## Conclusion

The findings of the present study indicated that the simplified Chinese IGDS-SF9, BSMAS, and SABAS had robust psychometric properties. Therefore, they are reliably and validly capable of assessing internet-related addictions among mainland Chinese children. These scales are, to date, the shortest and easiest to use instruments for assessing specific internet-related addictions. Furthermore, the items of all the three specific scales contributed significantly to the derived factor loadings. There were significant positive relationships between these scales and depression, anxiety, and stress as well as other variables such as time spent on these behaviors or activities demonstrating good construct validity.

## Data Availability Statement

The datasets generated for this study are available on request to the corresponding author.

## Ethics Statement

The studies involving human participants were reviewed and approved by the Ethics committee of the Hong Kong Polytechnic University (IRB ref: HSEARS20190718001) and the Institutional Review Board (IRB) of the Jiangxi Psychological Consultant Association (IRB ref: JXSXL-2019-J022). Written informed consent to participate in this study was provided by the participants’ legal guardian/next of kin.

## Author Contributions

I-HC, C-YL, and AP contributed conception and design of the study. I-HC organized the database. I-HC and C-YL performed the statistical analysis. DA wrote the first draft of the manuscript. I-HC, C-YL, and C-YC wrote sections of the manuscript. MG, C-YL, and C-YC critically commented on the draft. All authors contributed to the article and approved the submitted version.

## Conflict of Interest

The authors declare that the research was conducted in the absence of any commercial or financial relationships that could be construed as a potential conflict of interest.

## References

[1] Kumar M Mondal A A study on Internet addiction and its relation to psychopathology and self-esteem among college students. Ind Psychiatry J (2018) 27(1):61–6. 10.4103/ipj.ipj_61_17 PMC6198588 30416293

[2] De Berardis D D’Albenzio A Gambi F Sepede G Valchera A Conti CM Alexithymia and its relationships with dissociative experiences and Internet addiction in a nonclinical sample. CyberPsychol Behav (2009) 12(1):67–9. 10.1089/cpb.2008.0108 19132914

[3] De Berardis D Fornaro M Orsolini L Valchera A Carano A Vellante F Alexithymia and Suicide Risk in Psychiatric Disorders: A Mini-Review. Front Psychiatry (2017) 8:148. 10.3389/fpsyt.2017.00148 28855878 PMC5557776

[4] Samaha M Hawi NS Griffiths MD The Digital Addiction Scale for Children: Development and validation. Cyberpsychol Behav Soc Network (2019) 22:771–8. 10.1089/cyber.2019.0132 31755742

[5] Griffiths MD Szabo A Is excessive online usage a function of medium or activity? An empirical pilot study. J Behav Addict (2014) 3:74–7. 10.1556/JBA.2.2013.016 PMC4117278 25215216

[6] Griffiths MD Internet addiction-time to be taken seriously? Addict Res (2000) 8(5):413–8. 10.3109/16066350009005587

[7] Anand N Jain PA Prabhu S Thomas C Bhat A Prathyusha PV Internet Use Patterns, Internet Addiction, and Psychological Distress Among Engineering University Students: A Study from India. Indian J Psychol Med (2018) 40(5):458–67. 10.4103/IJPSYM.IJPSYM_135_18 PMC6149312 30275622

[8] Hasan AA-H Abu Jaber A The relationship between Internet addiction, psychological distress, and coping strategies in a sample of Saudi undergraduate students. Perspect Psychiatr Care (2019) 56(3):495–50. 10.1111/ppc.12439 31571247

[9] Saikia AM Das J Barman P Bharali MD Internet addiction and its relationships with depression, anxiety, and stress in urban adolescents of Kamrup District, Assam. J Family Community Med (2019) 26(2):108–12. 10.4103/jfcm.JFCM_93_18 PMC6515762 31143082

[10] Wang L Luo J Bai Y Kong J Luo J Gao W Internet addiction of adolescents in China: Prevalence, predictors, and association with well-being. Addict Res Theory (2013) 21(1):62–9. 10.3109/16066359.2012.690053

[11] Shao Y-J Zheng T Wang Y-Q Liu L Chen Y Yao Y-S Internet addiction detection rate among college students in the People’s Republic of China: a meta-analysis. Child Adolesc Psychiatry Ment Health (2018) 12:25–5. 10.1186/s13034-018-0231-6 PMC5970523 29849754

[12] Li L Xu D-D Chai J-X Wang D Li L Zhang L Prevalence of Internet addiction disorder in Chinese university students: A comprehensive meta-analysis of observational studies. J Behav Addict (2018) 7(3):610–23. 10.1556/2006.7.2018.53 PMC6426360 30010411

[13] American Psychiatric Association DSMTF Diagnostic and statistical manual of mental disorders: DSM-5. 5th ed American Psychiatric Association: Arlington, VA (2013).

[14] Leung H Pakpour AH Strong C Lin Y-C Tsai M-C Griffiths MD Measurement invariance across young adults from Hong Kong and Taiwan among three internet-related addiction scales: Bergen Social Media Addiction Scale (BSMAS), Smartphone Application-Based Addiction Scale (SABAS), and Internet Gaming Disorder Scale-Short Form (IGDS-SF9) (Study Part A). Addictive Behav (2020) 101:105969. 10.1016/j.addbeh.2019.04.027 31078344

[15] Yam C-W Pakpour AH Griffiths MD Yau W-Y Lo C-LM Ng JMT Psychometric testing of three Chinese online-related addictive behavior instruments among Hong Kong university students. Psychiatr Q (2019) 90(1):117–28. 10.1007/s11126-018-9610-7 30328020

[16] Griffiths MD Pontes HM Internet addiction disorder and internet gaming disorder are not the same. J Addict Res Ther (2014) 5(4):e124. 10.4172/2155-6105.1000e124

[17] Montag C Bey K Sha P Li M Chen Y-F Liu W-Y Is it meaningful to distinguish between generalized and specific Internet addiction? Evidence from a cross-cultural study from Germany, Sweden, Taiwan and China. Asia-Pacific Psychiatry (2015) 7(1):20–6. 10.1111/appy.12122 24616402

[18] Liang L Zhou D Yuan C Shao A Bian Y Gender differences in the relationship between internet addiction and depression: A cross-lagged study in Chinese adolescents. Comput Hum Behav (2016) 63:463–70. 10.1016/j.chb.2016.04.043

[19] Tao R Huang X Wang J Zhang H Zhang Y Li M Proposed diagnostic criteria for internet addiction. Addiction (2010) 105(3):556–64. 10.1111/j.1360-0443.2009.02828.x 20403001

[20] Zhang R-P Bai B-Y Jiang S Yang S Zhou Q Parenting styles and internet addiction in Chinese adolescents: Conscientiousness as a mediator and teacher support as a moderator. Comput Hum Behav (2019) 101:144–50. 10.1016/j.chb.2019.07.019

[21] Chen IH Strong C Lin Y-C Tsai M-C Leung H Lin C-Y Time invariance of three ultra-brief internet-related instruments: Smartphone Application-Based Addiction Scale (SABAS), Bergen Social Media Addiction Scale (BSMAS), and the nine-item Internet Gaming Disorder Scale- Short Form (IGDS-SF9) (Study Part B). Addictive Behav (2020) 101:105960. 10.1016/j.addbeh.2019.04.018 31072648

[22] Pontes HM Griffiths MD Measuring DSM-5 internet gaming disorder: Development and validation of a short psychometric scale. Comput Hum Behav (2015) 45:137–43. 10.1016/j.chb.2014.12.006

[23] Pontes HM Stavropoulos V Griffiths MD Measurement invariance of the Internet Gaming Disorder Scale–Short-Form (IGDS9-SF) between the United States of America, India and the United Kingdom. Psychiatry Res (2017) 257:472–8. 10.1016/j.psychres.2017.08.013 28837939

[24] Wu T-Y Lin C-Y Årestedt K Griffiths MD Broström A Pakpour AH Psychometric validation of the Persian nine-item Internet Gaming Disorder Scale-Short Form: Does gender and hours spent online gaming affect the interpretations of item descriptions? J Behav Addict (2017) 6(2):256–63. 10.1556/2006.6.2017.025 PMC5520122 28571474

[25] Pontes HM Griffiths MD Portuguese validation of the Internet Gaming Disorder Scale – Short-Form (IGD9-SF). Cyberpsychol. Behav Soc Network. (2016) 19:288–93. 10.1089/cyber.2015.0605 26974853

[26] Beranuy M Machimbarrena JM Vega MA Carbonell X Griffiths MD Pontes HM Spanish Validation of the Internet Gaming Disorder Scale-Short Form (IGDS9-SF): Prevalence and relationship with online gambling and quality of life. Int J Environ Public Health (2020) 17:1562. 10.3390/ijerph17051562 PMC7084394 32121280

[27] Pontes HM Macur M Griffiths MD Internet Gaming Disorder among Slovenian primary schoolchildren: Findings from a nationally representative sample of adolescents. J Behav Addict (2016) 5:304–10. 10.1556/2006.5.2016.042 PMC5387781 27363464

[28] Monacis L de Palo V Griffiths MD Sinatra M Validation of the Internet Gaming Disorder Scale-Short Form (IGDS9-SF) in an Italian-speaking sample. J Behav Addict (2016) 5(4):683–90. 10.1556/2006.5.2016.083 PMC5370374 27876422

[29] T’ng ST Pau K Validation of a translated Internet Gaming Disorder Scale (short form) and measurement invariance across sex groups in Malaysian samples. Curr Psychol (2020). 10.1007/s12144-020-00668-6

[30] Arıcak OT Dinç M Yay M Griffiths MD Adapting the short form of the Internet Gaming Disorder Scale into Turkish: Validity and reliability. Addicta: Turkish J Addict (2019) 5:629–35. 10.15805/addicta.2019.6.1.0027

[31] Evren C Dalbudak E Topcu M Kutlu N Evren B Pontes HM Psychometric validation of the Turkish nine-item Internet Gaming Disorder Scale–Short Form (IGDS9-SF). Psychiatry Res (2018) 265:349–54. 10.1016/j.psychres.2018.05.002 29793049

[32] Andreassen CS Billieux J Griffiths MD Kuss DJ Demetrovics Z Mazzoni E The relationship between addictive use of social media and video games and symptoms of psychiatric disorders: A large-scale cross-sectional study. Psychol Addictive Behav (2016) 30(2):252–62. 10.1037/adb0000160 26999354

[33] Griffiths MD A ‘components’ model of addiction within a biopsychosocial framework. J Subst Use (2005) 10(4):191–7. 10.1080/14659890500114359

[34] Bányai F Zsila Á Király O Maraz A Elekes Z Griffiths MD Problematic social media use: Results from a large-scale nationally representative adolescent sample. PLoS One (2017) 12(1), e0169839. 10.1371/journal.pone.0169839 28068404 PMC5222338

[35] Csibi S Demetrovics Z Szabo A Hungarian adaptation and psychometric characteristics of Brief Addiction to Smartphone Scale (BASS) [In Hungarian]. Psychiatria Hungarica (2016) 31(1):71–7. 27091924

[36] Monacis L de Palo V Griffiths MD Sinatra M Social networking addiction, attachment style, and validation of the Italian version of the Bergen Social Media Addiction Scale. J Behav Addict (2017) 6(2):178–86. 10.1556/2006.6.2017.023 PMC5520120 28494648

[37] Lin C-Y Imani V Broström A Nilsen P Fung XCC Griffiths MD Smartphone Application-Based Addiction among Iranian adolescents: A psychometric study. Int J Ment Health Addict (2019) 17(4):765–80. 10.1007/s11469-018-0026-2

[38] Csibi S Griffiths MD Cook B Demetrovics Z Szabo A The psychometric properties of the Smartphone Application-Based Addiction Scale (SABAS). Int J Ment Health Addict (2018) 16(2):393–403. 10.1007/s11469-017-9787-2 29670500 PMC5897481

[39] Altundağ Y Yandi A Ali ÜNAL Adaptation of Application-Based Smartphone Addiction Scale to Turkish cultures. Sakarya Univ J Educ (2019) 9(2):261–81. 10.19126/suje.516365

[40] Gökler ME Bulut YE Validity and reliability of the Turkish version of the Smart Phone Application Based Addiction Scale. J Cognitive-Behav Psychother Res (2019) 8(2):100–6. 10.5455/JCBPR.38288

[41] Soraci P Ferrari A Urso A Griffiths MD Psychometric properties of the Italian version of the Smartphone Application-Based Addiction Scale (SABAS). Int J Ment Health Addict (2020). 10.1007/s11469-020-00222-2 PMC5897481 29670500

[42] Sojević M Pećanac D Latas M Connection of depression, anxiety and impulsivity with the way of using modern mobile phones among students. Medicinski Podmladak (2018) 69(4):27–34. 10.5937/mp69-17929

[43] Lovibond PF Lovibond SH Manual for the Depression Anxiety Stress Scales. 2nd ed Sydney: Psychology Foundation (1995).

[44] Shaw T Campbell MA Runions KC Zubrick SR Properties of the DASS-21 in an Australian community adolescent population. J Clin Psychol (2017) 73(7):879–92. 10.1002/jclp.22376 27774593

[45] Wang K Shi H-S Geng F-L Zou L-Q Tan S-P Wang Y Cross-cultural validation of the Depression Anxiety Stress Scale–21 in China. Psychol Assess (2016) 28(5):e88–100. 10.1037/pas0000207 26619091

[46] Jette DU Warren RL Wirtalla C Functional independence domains in patients receiving rehabilitation in skilled nursing facilities: Evaluation of psychometric properties. Arch Phys Med Rehabil (2005) 86(6):1089–94. 10.1016/j.apmr.2004.11.018 15954045

[47] Cheng C-P Luh W-M Yang A-L Su C-T Lin C-Y Agreement of children and parents scores on Chinese Version of Pediatric Quality of Life Inventory Version 4.0: Further psychometric development. Appl Res Qual Life (2016) 11(3):891–906. 10.1007/s11482-015-9405-z

[48] Wang Y-S Wang H-Y Shee DY Measuring e-learning systems success in an organizational context: Scale development and validation. Comput Hum Behav (2007) 23(4):1792–808. 10.1016/j.chb.2005.10.006

[49] Bentler PM Bonett DG Significance tests and goodness of fit in the analysis of covariance structures. Psychol Bull (1980) 88(3):588–606. 10.1037/0033-2909.88.3.588

[50] Hu L Bentler PM Cutoff criteria for fit indexes in covariance structure analysis: Conventional criteria versus new alternatives. Struct Equation Model.: A Multidiscip J (1999) 6(1):1–55. 10.1080/10705519909540118

[51] Jafari P Nozari F Ahrari F Bagheri Z Measurement invariance of the Depression Anxiety Stress Scales-21 across medical student genders. Int J Med Educ (2017) 8:116–22. 10.5116/ijme.58ba.7d8b PMC5376494 28362630

[52] van den Brink W ICD-11 Gaming Disorder: Needed and just in time or dangerous and much too early?: Commentary on: Scholars’ open debate paper on the World Health Organization ICD-11 Gaming Disorder proposal (Aarseth et al.). J Behav Addict (2017) 6(3):290–2. 10.1556/2006.6.2017.040 PMC5700734 28033714

[53] Lin C-Y Luh W-M Cheng C-P Yang A-L Su C-T Ma H-I Measurement equivalence across child self-reports and parent-proxy reports in the Chinese Version of the Pediatric Quality of Life Inventory Version 4.0. Child Psychiatry Hum Dev (2013) 44(5):583–90. 10.1007/s10578-012-0352-8 23242709

[54] Lin C-Y Luh W-M Yang A-L Su C-T Wang J-D Ma H-I Psychometric properties and gender invariance of the Chinese version of the self-report pediatric quality of life inventory version 4.0: short form is acceptable. Qual Life Res (2012) 21(1):177–82. 10.1007/s11136-011-9928-1 21574017

[55] Chen FF Sensitivity of goodness of fit indexes to lack of measurement invariance. Struct Equation Model (2007) 14(3):464–504. 10.1080/10705510701301834

[56] Sigerson L Li AYL Cheung MWL Luk JW Cheng C Psychometric properties of the Chinese Internet Gaming Disorder Scale. Addictive Behav (2017) 74:20–6. 10.1016/j.addbeh.2017.05.031 28558336

[57] Lin C-Y Luh W-M Cheng C-P Yang A-L Ma H-I Evaluating the wording effect and psychometric properties of the kid-KINDL: Using the multitrait-multimethod approach. Eur J Psychol Assess (2014) 30(2):100–9. 10.1027/1015-5759/a000175

[58] Lin C-Y Broström A Nilsen P Griffiths M Pakpour A Psychometric validation of the Persian Bergen Social Media Addiction Scale using classic test theory and Rasch models. J Behav Addict (2017) 6(4):620–9. 10.1556/2006.6.2017.071 PMC6034942 29130330

